# MR1-Restricted Mucosal-Associated Invariant T Cells and Their Activation during Infectious Diseases

**DOI:** 10.3389/fimmu.2015.00303

**Published:** 2015-06-16

**Authors:** Lauren J. Howson, Mariolina Salio, Vincenzo Cerundolo

**Affiliations:** ^1^MRC Human Immunology Unit, Weatherall Institute of Molecular Medicine, University of Oxford, Oxford, UK

**Keywords:** MAIT cells, infection, MR1, bacteria, vitamins

## Abstract

MR1-restricted mucosal-associated invariant T (MAIT) cells recognize vitamin B metabolites, which are generated by a broad range of bacteria, from *Escherichia coli* to *Mycobacterium tuberculosis* and BCG. MAIT cells have been described as innate sensors of infection as they accumulate early in infected tissues. MAIT cells maintain an activated phenotype throughout the course of infections, secrete inflammatory cytokines, and have the potential to directly kill infected cells, playing an important role in shaping the host response. In this review, we will discuss the current knowledge regarding the molecular mechanisms that underline MAIT cells activation in sterile and non-sterile inflammatory conditions.

## Introduction

Innate-like lymphocytes, such as invariant natural killer T (iNKT) cells, have gained great interest since their discovery, as these cells lie at the interface between innate and adaptive immune responses. iNKT cells are memory cells bearing a semi-invariant T cell receptor (TCR) through which they recognize self- and bacterial-derived lipid antigens presented by CD1d molecules. During pathogen infection, iNKT cells facilitate adaptive immune responses by inducing dendritic cell maturation (1). Over the last few years, in addition to iNKT cells, another innate-like lymphocyte cell subset has been the focus of much research: mucosal-associated invariant T (MAIT) cells, recognizing a unique family of bacterial-derived metabolites in the context of the major histocompatibility complex (MHC) class I-like molecule MR1.

## MR1 Structure and Function

The MR1 gene was first described in 1995 in a genome screen aimed at identifying novel MHC-related genes (2). The human MR1 gene is located on chromosome 1, like CD1 genes, and despite a similar intron/exon organization to classical MHC molecules, it is non-polymorphic. MR1 is highly conserved among mammalian species, with 90% sequence homology between human and mouse MR1-ligand binding domains and a high level of functional cross-reactivity, a feature reminiscent of CD1 molecules (3, 4). Similar to MHC class I molecules, MR1 associates with β_2_-microglobulin and interacts with the peptide-loading complex during biosynthesis in the endoplasmic reticulum (5, 6). In 2003, the connection between MR1 and MAIT cells was made by demonstrating the central role of MR1, along with commensal flora and B cells, for MAIT cell development (7). Although MR1 is ubiquitously transcribed, its physiological expression at the cell surface has been difficult to demonstrate, with the exception of B cell subsets in the intestinal mucosa (8). A study of mouse MR1 reported that only the folded form of MR1 was able to activate MAIT cells and certain mutations in the putative binding groove disrupted MAIT cell activation, through presentation of yet unknown antigens (9). These results led to great interest in determining the identity of the antigens presented.

The observation that MAIT cells have anti-microbial activity provided important insights into the identification of the MAIT agonists presented by MR1. Kjer-Nielsen et al. (10) showed that, similar to other MHC class I and MHC class I-like molecules, MR1 was unable to fold correctly in refolding buffer alone. This suggested that it required the presence of its ligand in order to stabilize its structure. Refolding was attempted in a number of conditions using RPMI-1640 as a control. Surprisingly, it was found that MR1 molecules were able to fold in this cell culture media, which was known to contain a number of vitamins produced by microbes, but not present in mammals. This observation-based approach led Kjer-Nielsen and colleagues to test the ability of vitamins to bind and stabilize MR1, and ultimately led to the discovery of a new class of antigens: vitamin B metabolites (10). The first crystal structure of the MR1-ligand complex was with the folic acid (vitamin B9) metabolite 6-formylpterin (6-FP), which was shown to be unable to activate MAIT cells (10). To determine the identity of MAIT stimulatory ligands, MR1 molecules were refolded in the presence of the supernatant from *Salmonella typhimurium* bacterial cultures, as it was known that *Salmonella*-infected cells strongly activate MAIT cells (11, 12). By high-resolution mass spectrometry (MS) [ESI–TOF-MS], MR1 was shown to associate with a compound at *m/z* 329.1100, with an atomic composition matching that of several riboflavin (vitamin B2) derivatives: 6,7-dimethyl-8-d-ribityllumazine (RL-6,7-diMe), 7-hydroxy-6-methyl-8-d-ribityllumazine (RL-6-Me-7-OH), and reduced 6-hydroxymethyl-8-d-ribityllumazine (rRL-6-CH_2_OH). In functional assays, these compounds were stimulatory and induced CD69 expression as well as interferon-gamma (IFNγ) and tissue necrosis factor-alpha (TNFα) production by MAIT cells (10).

In order to understand the origin of the above MAIT cell ligands, *Lactocossus lactis* mutants of the four-gene operon, which controls riboflavin biosynthesis, were studied (13). These results confirmed that riboflavin is necessary and sufficient to generate natural MAIT cell ligands. Furthermore, these results pointed to the compound 5-amino-6-d-ribitylaminouracil (5-A-RU) as an intermediate necessary for MAIT cell activation. This intermediate does not bind to MR1, but forms MAIT-stimulating ligands through a non-enzymatic condensation with glyoxal or methylglyoxal species, which can be of bacterial or host cell origin (such as byproducts of glycolysis). The yields of these condensation reactions are the unstable, yet potent intermediates 5-(2-oxoethylideneamino)-6-d-ribitylaminouracil (5-OE-RU) and 5-(2-oxopropylideneamino)-6-d-ribitylaminouracil (5-OP-RU). These molecules can be captured and stabilized through Schiff bases with Lys43 in the MR1 groove. Furthermore, these compounds are the substrates for conversion to RL-6,7-diMe. Mass spectrometry analysis of MR1 refolded in the presence of culture supernatants from *S. typhimurium, E. coli* (DH5α), or rRL-6-CH_2_OH also revealed species with matching properties to the synthetic 5-OP-RU, raising the possibility that the compound initially identified as MR1 ligand was indeed 5-OP-RU (10, 13). These results provide evidence that MAIT cells are able to sense a wide range of bacteria through detection of vitamin metabolites, including transitory intermediates, presented by MR1 molecules.

## MAIT Cell Phenotype

Mucosal-associated invariant T cells were identified based on their surface phenotype and mucosal tissue localization. The MAIT cell invariant TCR Vα7.2–Jα33/12/20 in humans was first described in 1993 as one of the few preferentially used TCRs in the double-negative T cell compartment (14). This finding was the first evidence of a new subset of T cells possibly recognizing a limited set of antigens in the context of non-polymorphic antigen presenting molecules. It was not until 1999 that the MAIT cell subset was defined as a conserved subpopulation distinct from MHC class I- and CD1-restricted cells with an activated/memory phenotype (15). They also reported that the human MAIT cell TCR β chain usage was primarily TRBV6 or TRBV20. Initial research into MAIT cells has been hampered by the lack of specific reagents; however, the generation of a monoclonal antibody specific for the Vα7.2 TCR chain (16) and MR1 tetramers (17) have recently enabled the functional and phenotypic analysis of MAIT cells and brought them to the forefront of innate-like lymphocyte research. MAIT cells are defined as CD3^+^ Vα7.2^+^ CD161^++^ and either CD8^+^ or double-negative T cells (12, 16). MR1-loaded tetramer experiments have also identified a small subset of MAIT cells that are CD4^+^ (17). All human MAIT cell subsets express the transcription factor PLZF, known to direct the effector program of the iNKT cell lineage (18). However, murine MAIT cells do not express PLZF (16), thus the functional relevance of this observation is currently unclear. MAIT cells can also be defined based on co-expression of interleukin (IL)-18R (12) and CD26 (19). Furthermore, in adults, peripheral blood MAIT cells have an effector memory phenotype defined as CD45RO^+^, CD62L^lo^, CD95^hi^ CD122^int^, CD127^int^, and they express tissue-homing chemokine receptors: CCR5, CCR6, CXCR6, and CCR9 (20). By contrast, MAIT cells do not express CCR7 that is a marker for homing to lymph nodes. The distinct memory phenotype and peripheral location of these cells are linked to their unique developmental pathway.

## MAIT Cell Development

Mucosal-associated invariant T cells develop and undergo selection in the thymus. Like iNKT cells, MAIT cells are selected by CD4/CD8 double-positive (DP) thymocytes (21). MR1 expression on DP thymocytes is essential, as MR1-deficient mice do not develop T cells expressing the MAIT cell TCR at detectable levels (7). Although thymic selection occurs independently of B cell and commensal flora (16), B cells are essential for MAIT cell peripheral expansion and memory phenotype acquisition. However, the endogenous antigen(s) capable of selecting MAIT cells in the thymus to date remain unclear. Furthermore, unlike iNKT cells, human MAIT cells egress the thymus as naïve cells, that in the periphery acquire a memory phenotype prior to birth (22).

In mice, the interaction of MAIT cells with B cells in the periphery drives the acquisition of the MAIT cell memory phenotype (16). In humans, a recent study analyzed MAIT cell development in second trimester fetal tissues, and reported acquisition of innate-like anti-microbial activity already *in utero* (22). This study suggested that MAIT cell maturation begins in the secondary lymphoid organs, where they reported Vα7.2^+^ CD161^+^ cells acquire expression of the transcription factor PLZF, CD62L, and CD45RO. In fetal liver and mucosal tissues, Vα7.2^+^ CD161^+^ cells expressed high levels of PLZF, IL-18Rα, CD62L, and CD45RO and were capable of responding to bacterial antigen stimulation both through proliferation and production of IFNγ and IL-22. While murine studies have revealed the importance of the microbiota in shaping MAIT cell differentiation, as they are absent in germ-free mice (7), this study suggests that human MAIT cells develop prenatally, before establishment of the commensal microflora, although the factors driving this maturation remain to be identified (22). However, it remains unclear whether Vα7.2^+^ CD161^+^ T cells described by Leeansyah and colleagues are MR1-restricted MAIT cells, as MR1-tetramer staining or MR1-blocking antibody experiments were not performed (22).

The interaction of MR1 expressing B cells and MAIT cells in the periphery is necessary for MAIT cell expansion in mice (16). Likewise in humans, individuals with mutated Bruton’s tyrosine kinase, and thus with disrupted B cell development, have reduced MAIT cell TCR transcripts in the blood compared to healthy controls (7). These results are consistent with the observation that both primary B cells and B cell lines can efficiently present bacterial antigens to MAIT cells in an MR1-dependent manner (23).

Together, the results from these studies suggest that MAIT cells are selected and undergo maturation prior to exposure to commensal bacteria. It can be speculated that peripheral expansion of MAIT cells may occur via presentation of an endogenous antigen present at mucosal sites or through maternally derived commensal flora antigen, possibly carried in the amniotic fluid.

## MAIT Tissue Distribution

Mucosal-associated invariant T cells are primarily located in the mucosal compartments and blood. The expression of chemokine receptors CXCR6 and CCR9 at the surface of MAIT cells suggested that they traffic to the tissues, particularly the intestine, lung, and liver (20). Indeed, these tissues have a higher proportion of MAIT cells, composing 20–40% of liver T cells and 4–10% intestinal T cells (20, 24). MAIT cells compose 1–8% of T cells in the blood. Analysis of the Vα7.2–J33 and Vα7.2–Jα12 transcripts in different tissues revealed that MAIT cells are also present in the kidney and at a lower frequency in the tonsils and lymph nodes (25). They also reported variable expression of these transcripts in the prostate and ovary (25). Analysis of the α chains revealed a predominance of Vα7.2–Jα33 in the blood and Vα7.2–Jα12 in tissues indicating that these two MAIT subsets might differ in their tissue-homing properties (25). Further investigation is needed to determine whether these two subtypes are functionally distinct in terms of their antigen/pathogen specificity or are related to the infection history of the individual.

In humans, circulating MAIT cells are more abundant than iNKT cells, while in mice they are far fewer (15, 16, 20). Studies assessing the influence of age and gender of MAIT cells found that MAIT cells decrease with age, but are not significantly different between males and females (26–28). The MAIT cell phenotype also changes according to the tissue compartment. For example, liver MAIT cells have a more activated phenotype compared to blood MAIT cells and express higher levels of CD69, CD38 and HLA-DR, possibly reflecting continuous antigen exposure (24).

## MR1-Dependent and -Independent MAIT Cell Activation

In 2010, two publications described a wide range of phylogenetically diverse bacteria and yeast that were able to activate MAIT cells. These microbes included: *E. coli*, *Pseudomonas aeruginosa*, *Klebsiella pneumoniae*, *Lactobacillus acidophilus*, *Staphylococcus aureus, Staphylococcus epidermidis, Saccharomyces cerevisiae, Candida glabrata*, and *Candida albicans* (11, 12). Conversely, viruses and few bacterial species such as *Enterococcus faecalis* and *Streptococcus* group A did not elicit MR1-dependent MAIT cell activation. The later discovery of the MAIT cell ligand offered the explanation for the lack of response as these bacteria lack the vitamin B metabolites that bind MR1 molecules and stimulate MAIT cells (10).

### Cytokine production

**Figure 1:**
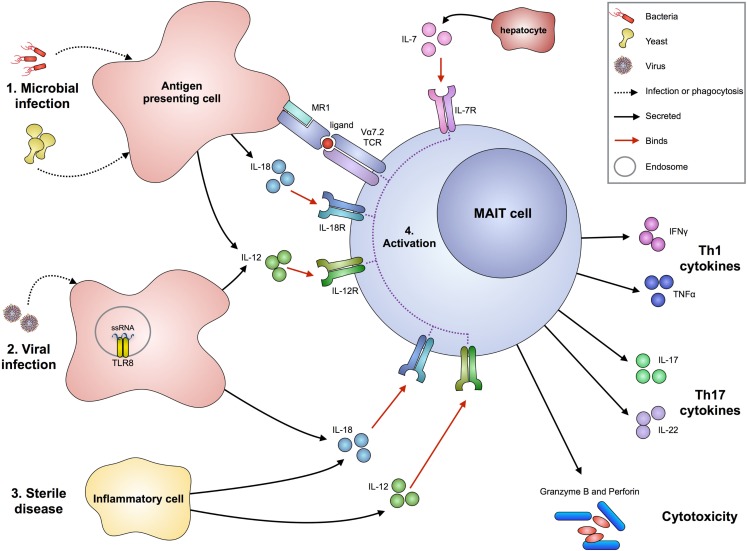
**Proposed mechanisms of MAIT cell activation in infection and sterile disease**. Bacteria or yeast (1) can stimulate MAIT cell activation following infection or phagocytosis by antigen presenting cells. These cells can then present microbial-derived vitamin B metabolites via MR1 (associated with β2-microglobulin) to the Vα7.2-bearing MAIT TCR. Upon infection, antigen presenting cells also produce IL-12 and IL-18 cytokines that can activate MAIT cells in an antigen-independent mechanism. The production of IL-7 from hepatocytes can act synergistically to enhance MAIT cell activation. Viruses (2) can stimulate MAIT cells through detection of their molecular patterns by pattern recognition receptors, such as ssRNA by TLR8 on antigen presenting cells, resulting in the production of IL-12 and IL-18. In sterile disease (3), such as autoimmunity, cells that pathologically express cytokines IL-12 and IL-18 can activate MAIT cells. The activation of MAIT cells (4) results in the production of Th1 cytokines IFNγ and TNFα, Th17 cytokines IL-17 and IL-22 (particularly by small intestine-derived MAIT cells), and release of perforin and granzyme B to directly kill infected cells.

Mucosal-associated invariant T cells can produce pro-inflammatory cytokines upon activation (Figure 1). Stimulation with phorbol 12-myristate 13-acetate (PMA) and ionomycin, as well as bacteria, showed that human MAIT cells are capable of producing IFNγ, TNFα, IL-2, and IL-17 but not IL-10 (20, 24). *Ex vivo* multiparametric mass cytometry in three donors recently confirmed that there are distinct subsets of circulating MAIT cells releasing different cytokine combinations, most frequently MIP1β, IFNγ, TNFα, and IL-2, and to a lesser extent IL-4 and IL-10 (25). Furthermore, MAIT cells from the small intestine are capable of producing IL-22 in response to *E. coli*, but this was not observed in MAIT cells from the liver or lung (22). Thus, MAIT cells have a mixed Th1/Th17 cytokine profile with differences according to their peripheral location, possibly reflecting local environmental imprinting.

### Cytotoxicity

An important property of MAIT cells is their ability to kill infected cells (Figure 1). Cytotoxicity occurs primarily via granule exocytosis and relies on the effector cell containing components that enable destruction of a target cell. These include perforin and granzymes that, in humans, consist of five isoforms with different substrate specificities: granzyme (Gr) A, GrB, GrH, GrK, and GrM (29). GrB and perforin have been directly linked to the cytotoxic activity of CD8^+^ T cells (30). It has been shown that MAIT cells are able to lyse bacteria-infected epithelial cells (31) and *E. coli-*infected THP1 cells (32) in an MR1-dependent manner. Interestingly, human MAIT cells have a unique cytotoxic profile, characterized by low perforin expression, but high GrA and GrK, contained in CD107^+^ lytic granules (32). Upon antigen recognition, an MR1-dependent GrB upregulation and an MR1-independent perforin upregulation was observed, suggesting that antigen pre-exposure licenses MAIT to acquire a stronger cytotoxic phenotype (32). The ability to kill infected target cells suggests that MAIT cells could have an important role in controlling infections by intracellular bacteria, such as *Shigella* and *Salmonella*, although the latter may escape MAIT cell detection by preventing vacuole–lysosomal fusion (33). Indeed, it has been shown that, while HeLa cells infected with *Shigella flexneri* can be readily killed by MAIT cells, HeLa cells infected with *S. typhimurium* failed to be detected or lysed by MAIT cells (31).

### MR1-independent activation

Like other innate-like cells, MAIT cells can also respond to infection in an MR1-independent manner. For example, the *in vitro* MAIT cell response to cells infected with *Mycobacterium bovis* Bacillus Calmette–Guérin (BCG) is largely MR1-independent (34). The high expression of IL-18R on MAIT cells suggested that IL-18 could be one of the cytokines able to activate MAIT cells (20, 35). Therefore, cytokine-dependent activation of MAIT cells was explored in more detail and it was reported that MAIT cells could produce IFNγ when their TCR is blocked and they are cultured with IL-18 and IL-12 (36). Furthermore, cytokines can synergize with TCR stimulation to enhance MAIT cell activation. For example, liver-resident MAIT cells express high levels of IL-7R and IL-7 potentiates TCR-dependent secretion of Th1 cytokine and of IL-17A (24). These results suggest that multiple factors could shape MAIT cells responsiveness during bacterial infection, and their ability to respond to cytokine stimulation, independent of TCR cross-linking, may account for their response to microbes not containing MR1 ligands, such as viruses and some bacterial species (Figure 1).

## MAIT Cells in Bacterial Infections

Murine infection models and natural human infections have been researched in order to further our understanding of MAIT cells and their activation during pathological conditions.

In a mouse model of lung infection with live *Francisella tularensis* (LVS), it was demonstrated that MAIT cells colonize the lung in both early and intermediate phases of the infection, reach their peak expansion in the late clearance phase of infection, and persist following clearance of the bacteria (37). From day 8 of infection onward, MAIT cells in the lung produced anti-bacterial cytokines (IFNγ, TNFα, and IL-17A). The evidence for the importance of MAIT cells in controlling infection came from MR1 knockout mice, which showed a delay in clearance of the bacteria in the lungs (but not in the spleen or liver) and delayed appearance of adaptive immune responses in the lung (37). A similar increase in bacterial load in comparison with wild type mice was found when mice lacking MAIT cells were infected with *K. pneumonia* or *M. bovis* BCG (34, 38). Together, these studies indicate that the role of MAIT cells in controlling infection is not redundant, and is vital for timely mounting of effective adaptive immune responses.

### Tuberculosis

In humans, the contribution of MAIT cells to anti-bacterial immunity has been studied mostly during *Mycobacterium tuberculosis* infection. MAIT cells have been shown to be able to respond *in vitro* to *M. tuberculosis* infected primary lung epithelium and dendritic cells (11, 39). In individuals with active *M. tuberculosis* infection, it was found that circulating MAIT cells were in lower absolute numbers and proportions compared to healthy individuals (11, 12). This suggested that MAIT cells may migrate to inflamed tissues, where they can have a localized response to infection. There is also evidence that MAIT cells are enriched in the lung of healthy donors and therefore could respond early during the course of infection (11).

To assess whether the reduced number of MAIT cells is accompanied by an altered functionality, a study analyzed the cytokine production by MAIT cells from individuals with tuberculosis (TB) in response to different stimuli. Despite lower frequencies, a higher proportion of MAIT cells in individuals with active TB secreted IFNγ and TNFα in response to BCG stimulation as compared to healthy controls (although the overall response was very low) (40). More convincingly, a lower proportion of MAIT cells responded to *E. coli* in the same cohort of infected individuals compared to healthy controls. These results suggest that there may be an overall enrichment of MAIT cells specific for BCG compared to *E. coli* upon *in vivo* antigenic exposure.

This concept of expansion of pathogen-specific MAIT T cell clones is suggested by a recent paper reporting that heterogeneity in the TCR β chain usage by MAIT cell clones was directly related to their ligand specificity, and thus, to responses to different microbes (41). While this is a very attractive possibility, this observation requires further confirmation as the MAIT cells assessed in the above study were defined as Vα7.2^+^ CD8^+^ cells that produced TNFα in response to bacterial stimulation. Without using MAIT specific markers, such as CD161, IL-18R, or CD26, this strategy could have included a proportion of conventional Vα7.2^+^ T cells. Furthermore, no MR1 blocking experiments were included to demonstrate differential microbe recognition. Nevertheless, a contribution of the TCRβ chain sequence to antigen binding has been also suggested by a second study (42), where six MAIT TCRs with differing β chains were refolded and their biophysical parameters were measured. This study showed that the CDR3β loop usage directly impacts MAIT cell recognition of ligands through altering the TCR flexibility and contact with MR1 and the ligand (42); thus, suggesting a role of the TCR CDR3β loop in fine-tuning MAIT cells’ specificity.

These findings open up an exciting area of research, suggesting that this innate-like lymphocyte subset may be capable of forming memory-like responses upon selective expansion of MAIT cell clones following lifetime microbe exposure. The ability to form memory responses could have implications for the therapeutic use of MAIT cells.

### Other bacterial infections

Decreased numbers of circulating MAIT cells have also been reported in individuals with cystic fibrosis during infection with the opportunistic pathogen *P. aeruginosa* (43). In these individuals, the lower MAIT cell numbers also correlated with lung disease severity, systemic inflammation, and clinical status. However, whether the reduction in numbers was a consequence of their localization to the airway mucosa, or by itself influenced colonization and disease progression, was not addressed.

The relevance of MAIT cells in a model of invasive enteric infection was addressed in a study investigating the efficacy of an attenuated strain of *Shigella dysenteriae* (31). A specific reduction in circulating MAIT cells was observed at day 11 in subjects who received bacteria compared to controls. Individuals who responded to the vaccine, producing a specific IgA response, also had a trend toward a higher baseline proportion of MAIT cells and at day 11 had an increased proportion of activated MAIT cells as determined by HLA-DR upregulation.

Mucosal-associated invariant T cells have also been investigated in cases of severe infection with *Vibrio cholerae*. It was reported that MAIT cells activation, as measured by CD38 expression, peaks 7 days after the onset of infection (44). In adults, the proportion of MAIT cells did not change upon infection, but interestingly, in children a significant and persistent decrease in MAIT cell proportion was observed. The reason for the adult/child discrepancy is not clear and requires further investigation. Although *V. cholerae* is known to have a riboflavin biosynthetic pathway, *in vitro* experiments aimed at demonstrating the role of vitamin B2 metabolites in this recognition were not performed.

A specific decrease in MAIT cell counts (but not iNKT or γδ T cells) was also observed in severe infections. Critically ill patients admitted to the intensive care unit (ICU) with sepsis had lower circulating levels of MAIT cells compared to healthy controls and non-septic critically ill individuals (45). Patients with lower MAIT numbers were also more susceptible to hospital-acquired infections. Of interest, it was reported that streptococcal infections (which do not stimulate MAIT cells *in vitro*) induced a less pronounced decrease in MAIT cell numbers than non-streptococcal infections, although there was a large overlap between groups that prevented statistical significance.

## MAIT Cells in Viral Infection

Although it has been established that virally infected cells do not directly activate MAIT cells (12), several publications have investigated the numbers and activation of MAIT cells in human immunodeficiency virus (HIV) infection. The rationale behind investigating MAIT cells in the context of HIV is due to the effects this virus has on increasing the permeability of intestinal epithelia. This alteration causes translocation of microbial products from the gastrointestinal tract, resulting in systemic immune activation (46, 47).

### Human immunodeficiency virus

Two groups reported that circulating MAIT cells are reduced early in HIV infection compared to healthy controls (48, 49). This trend was also observed in individuals with chronic HIV, and numbers could not be restored following anti-retroviral therapy (49). Consistent with these findings, staining with MR1 tetramers loaded with 5-OP-RU confirmed that the tetramer-positive MAIT population is reduced during HIV infection (50).

The activation and function of circulating MAIT cells in HIV were also assessed, and it was found that they express lower levels of CD69 and produce less IFNγ and TNFα in response to *ex vivo* bacterial stimulation compared to healthy controls (49). Interestingly, after anti-retroviral therapy, a partial restoration of cytokine production was observed. However, this impaired activation and function of MAIT cells were not confirmed in a separate study (50).

The authors also investigated MAIT cells in the rectal and colon mucosa of HIV infected individuals, which were reduced, like circulating MAIT cells. However, mucosal numbers were better preserved and could be restored after anti-retroviral therapy (49, 51).

The decline and possible impaired activation of MAIT cells in patients infected with HIV are likely to affect the MAIT cells’ ability to control bacteria and yeast infections and, as a result, leave the host extremely vulnerable and contributing to acquired immune deficiency syndrome (AIDS) pathogenesis.

### Hepatitis B and C

As previously discussed, a large proportion of liver-resident T cells are MAIT cells. These liver-resident immune cells are of great interest as viruses, such as hepatitis B virus (HBV) and hepatitis C virus (HCV), can establish persistent infections in the liver. To determine whether liver MAIT cells could respond to innate signals upon viral infection, Toll-like receptor (TLR) agonists were used to stimulate liver intra-sinusoidal cells. It was found that TLR8 agonist ssRNA40 was able to activate liver MAIT and NK cells to produce large amounts of IFNγ (52). The response was MR1-independent and driven by IL-12 and IL-18 released by TLR8-stimulated monocytes. A similar activation of hepatic MAIT cells was observed in individuals infected with HBV and HCV. This preservation of anti-viral activity of MAIT cells in diseased livers could provide a possible therapeutic target for the treatment of viral infection.

## MAIT Cells in Sterile Disease

The capacity of MAIT cells to respond to cytokine stimulation, as described above, raises the possibility that these cells may also play a role in sterile inflammation such as autoimmune disorders and cancer.

### Multiple sclerosis (MS)

The role of MAIT cells in MS has been extensively researched. MAIT cells’ TCR was identified in central nervous system MS lesions using single-strand conformation polymorphism analysis in autoptic specimens (53). Normal frequency of MAIT cells was found in the blood of MS patients and MAIT cells were also detected in the cerebrospinal fluid of relapsing patients. This suggested a role for MAIT cells in the progression of this disease, which was functionally explored by a study using a mouse model for MS, experimental autoimmune encephalitis (EAE). In transgenic mice overexpressing the MAIT cell TCR, MAIT cells protected against EAE through suppression of Th1 cytokine production and increased production of IL-10 primarily by B cells (54). It was also observed that, in MR1 knockout mice, EAE was exacerbated, suggesting a new regulatory role for MAIT cells.

Conflicting results have emerged from studies assessing the distribution of circulating MAIT cells in MS patients. A study of an Italian cohort assessing MAIT cells of monozygotic twins with discordant disease states showed a greater number of circulating CD8^+^ CD161^++^ cells in MS patients compared to their healthy twin (55). By contrast, a study of a Japanese cohort showed decreased circulating MAIT cells in MS patients compared to healthy controls (56). This discrepancy between the two studies findings underlines the difficulties of studies with human subjects and suggests the variation in the commensal flora or lifetime infection burden may have affected MAIT cell frequencies in the two populations.

### Psoriasis

It has previously been reported that IL-17A is a vital component in the etiology of psoriasis (57). As MAIT cells are able to produce this cytokine, it was thought that they might be the CD8^+^ cells previously observed in psoriatic lesions. It was found that MAIT cells are indeed present in the psoriatic lesions along with Th17 cells (58). This provided evidence that MAIT cells may contribute to psoriasis, however, their activation in this setting is yet to be explored.

### Rheumatic diseases

Mucosal-associated invariant T cells have also been studied in the context of rheumatic diseases. It was found that individuals with systemic lupus erythromatosis (SLE) and rheumatoid arthritis (RA) had lower circulating levels of MAIT cells (59). Furthermore, MAIT cells have been detected in the synovial fluid of RA patients in higher proportions than in the blood. Individuals with SLE had MAIT cells with impaired activation, as they had a lower production of IFNγ when challenged with bacteria or stimulated with PMA and ionomycin. This impaired function was more prominent in individuals with lower MAIT cell numbers, suggesting a correlation between MAIT cell numbers and function. Conversely, in RA patients, the IFNγ secretion was preserved. To assess whether the inhibitory molecule PD-1 correlated with MAIT cell dysfunction, its expression on MAIT cells was assessed and found to be higher on MAIT cells in individuals with SLE compared to individuals with RA or healthy controls. However, after PD-1 blockade, MAIT cell activation was only partially restored, indicating other negative regulation mechanisms may be contributing to MAIT cell dysfunction in SLE.

### Inflammatory bowel disease and celiac disease

The location of MAIT cells in the small intestine mucosa makes them of great relevance in the pathogenesis of intestinal inflammatory disorders, such as inflammatory bowel diseases (IBD): Crohn’s disease and ulcerative colitis. A study reported that the number of circulating MAIT cells was reduced in individuals with Crohn’s disease and ulcerative colitis compared to healthy controls (60). A similar observation was reported with circulating MAIT cell numbers in individuals with celiac disease (61). It was also reported that MAIT cells were higher in number in inflamed compared to healthy tissues (60). Functional assessment of MAIT cells from individuals with IBD showed increased IL-17 production upon stimulation along with increased IL-22 in ulcerative colitis but decreased IFNγ secretion in individuals with Crohn’s disease. This first demonstration of activated MAIT cells in inflamed gut tissues warrants further investigation as to their potential pathogenic role.

### Cancer

A less explored area is the potential role of MAIT cells in cancer. A publication has suggested MAIT cells may be present in renal and brain cancers through detection of their TCR in these tissues (62). This presence correlated with the presence of pro-inflammatory cytokines in the tumor tissue, suggesting they could have anti-neoplastic functions. However, this has not been explored any further.

## MAIT Cell as a Potential Therapeutic Target

With their ability to alter the cytokine microenvironment to pro-inflammatory, direct killing of infected cells, potential to enhance adaptive immune responses, and the recent discovery of their activating ligands, MAIT cells may be an ideal target to manipulate for enhancing immune responses. An understanding of the potential of these cells could aid the designing of more targeted vaccines and effective immunotherapies.

The use of bacteria as adjuvants to enhance immune responses was first explored in 1891 when William Coley conducted intratumoral injections of *S. pyogenes* and *Serratia marcescens* to cause inflammation and subsequent destruction of the tumor cells (63). This form of immunotherapy, in which bacteria are used to stimulate anti-tumor immune responses, has not been highly successful with the notable exception of the intra-vesical instillation of BCG, which is currently the gold standard treatment of non-invasive bladder cancer. Although successful, it is still not well understood what the immune principles are that underlie its success. It has previously been discussed that MAIT cells are able to respond to BCG-infected cells *in vitro* (34, 40). Understanding whether MAIT cells are key players in mediating the success of BCG immunotherapy may help in designing more targeted therapies and also opens up the potential for MAIT cell-targeted immunotherapies for other cancers.

## Outstanding Questions

A number of questions still need to be addressed in the field of MAIT cell research. First, the full spectrum of MAIT-activating ligands remains to be explored. Whether other possible vitamin metabolites or small organic compounds could bind MR1 and stimulate MAIT cell responses is still an open question. Also, a priority will be identification of endogenous ligand(s) involved in shaping MAIT cell selection and maturation during development.

Second, although MAIT cells can respond to yeast species *in vitro*, the role of MAIT cells during yeast infections *in vivo* remains to be addressed.

Third, it is still not clear what factors determine the relative contribution of antigen-dependent versus cytokine-dependent MAIT cell response during an infection. Early findings suggest that the presence of the ligand, mode of infection, and subcellular localization of the bacteria may all impact the way MAIT cells respond during infection.

Finally, it is still not known whether the MAIT cell subset could be an effective therapeutic target. The identification of potent antigens for MAIT cells could provide a possible adjuvant in vaccine and immunotherapies targeting both infections and also non-sterile diseases.

## Concluding Remarks

The role of MAIT cells during infection is only beginning to be established, with their early infiltration into infected tissues, production of pro-inflammatory cytokines, and role in assisting an effective adaptive immune response. The effector functions of MAIT cells in sterile disease conditions are also being explored. Such an integral role in the progression of infection, and possibly sterile diseases, makes these cells an ideal target for enhancing immune responses.

## Conflict of Interest Statement

The authors declare that the research was conducted in the absence of any commercial or financial relationships that could be construed as a potential conflict of interest.
